# Pyruvate dehydrogenase levels are low in sepsis

**DOI:** 10.1186/cc14113

**Published:** 2015-03-16

**Authors:** E Nuzzo, X Liu, K Berg, L Andersen, M Doninno

**Affiliations:** 1 Beth Israel Deaconess Medical Center, Boston, MA, USA

## Introduction

Pyruvate dehydrogenase (PDH) is a key component of aerobic metabolism. Multiple rodent studies have shown that PDH levels are low in sepsis. This leads to a shift to anaerobic metabolism, resulting in increased lactic acid. Alteration in PDH levels during sepsis, however, has never been studied in humans. The aim of this study was to identify whether PDH levels (activity and quantity) were altered in humans in sepsis.

## Methods

We conducted a case-control study at a single urban tertiary care center. We compared PDH levels between sepsis and healthy control subjects by measuring PDH levels in peripheral blood mononuclear cells via a novel assay. We measured PDH levels in control subjects at baseline and in sepsis subjects at 0, 24, 48 and 72 hours.

## Results

**Figure 1:**
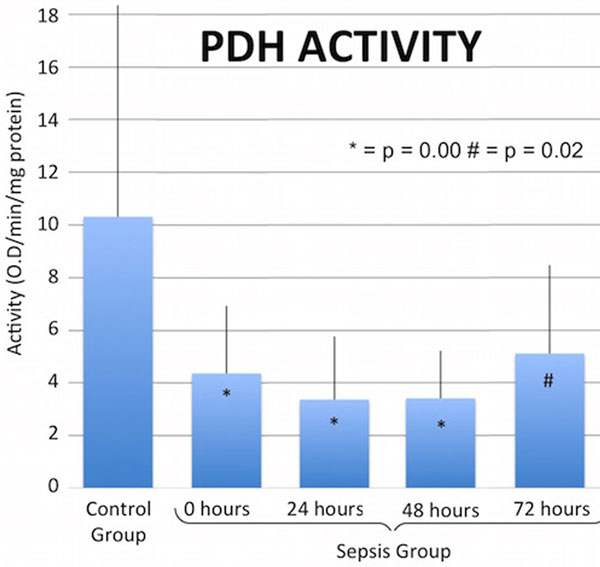


**Figure 2:**
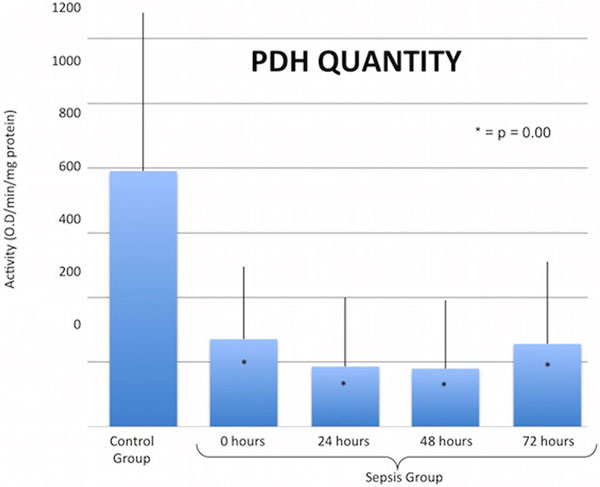


There were 39 sepsis (age 67 ± 14 years, M ± SD) and 19 control (age 50 ± 12 years) subjects of similar gender (56% and 63% female, respectively) and race (79% and 68% Caucasian, respectively). PDH levels in the sepsis group were significantly lower than the control group at all time points (Figures 1 and 2). After controlling for age, gender, race, and assay plate via multivariable linear regression, the effect of treatment group remained significant. We were unable to control for comorbid illness, which was exclusively concentrated in the sepsis group.

## Conclusion

PDH levels are significantly lowered in humans during sepsis when compared with healthy controls, even when controlling for age, race and gender. Further research is needed to determine whether this finding persists after adjustment for comorbid disease, and whether lower PDH levels are associated with clinical outcomes.

